# Lithocholic bile acid selectively kills neuroblastoma cells, while sparing normal neuronal cells

**DOI:** 10.18632/oncotarget.338

**Published:** 2011-10-11

**Authors:** Alexander A. Goldberg, Adam Beach, Gerald F. Davies, Troy A. A. Harkness, Andréa LeBlanc, Vladimir I. Titorenko

**Affiliations:** ^1^Department of Biology, Concordia University, Montreal, Quebec H4B 1R6, Canada; ^2^Department of Anatomy and Cell Biology, University of Saskatchewan, Saskatoon, Saskatchewan S7N 5E5, Canada; ^3^Department of Neurology and Neurosurgery, McGill University, Montreal, Quebec H3A 2B4, Canada; ^4^The Bloomfield Centre for Research in Aging, Lady Davis Institute for Medical Research, Jewish General Hospital, Montreal, Quebec H3T 1E2, Canada

**Keywords:** age-related diseases, cancer, neuroblastoma, breast cancer, glioma, anti-cancer drugs, apoptosis, bile acids, lithocholic acid

## Abstract

Aging is one of the major risk factors of cancer. The onset of cancer can be postponed by pharmacological and dietary anti-aging interventions. We recently found in yeast cellular models of aging that lithocholic acid (LCA) extends longevity. Here we show that, at concentrations that are not cytotoxic to primary cultures of human neurons, LCA kills the neuroblastoma (NB) cell lines BE(2)-m17, SK-n-SH, SK-n-MCIXC and Lan-1. In BE(2)-m17, SK-n-SH and SK-n-MCIXC cells, the LCA anti-tumor effect is due to apoptotic cell death. In contrast, the LCA-triggered death of Lan-1 cells is not caused by apoptosis. While low concentrations of LCA sensitize BE(2)-m17 and SK-n-MCIXC cells to hydrogen peroxide-induced apoptotic cell death controlled by mitochondria, these LCA concentrations make primary cultures of human neurons resistant to such a form of cell death. LCA kills BE(2)-m17 and SK-n-MCIXC cell lines by triggering not only the intrinsic (mitochondrial) apoptotic cell death pathway driven by mitochondrial outer membrane permeabilization and initiator caspase-9 activation, but also the extrinsic (death receptor) pathway of apoptosis involving activation of the initiator caspase-8. Based on these data, we propose a mechanism underlying a potent and selective anti-tumor effect of LCA in cultured human NB cells. Moreover, our finding that LCA kills cultured human breast cancer and rat glioma cells implies that it has a broad anti-tumor effect on cancer cells derived from different tissues and organisms.

## INTRODUCTION

Due to a multistep nature of the tumorigenesis process whose progression and completion requires an extended period of time, incidence rates of many cancers increase with age [1-3]. Therefore, cancer is considered as a disease associated with aging [3-7]. In the generally accepted paradigm of the relationship between aging and cancer, they share common aetiology (*i.e.*, an age-related progressive accumulation of cellular damage) and have coalescent mechanisms [4-9]. A body of evidence supports the validity of this paradigm. First, aging and cancer indeed have convergent underlying mechanisms. They include: 1) a longevity-defining signaling network that integrates the pro-aging AMP-activated protein kinase/target of rapamycin (AMPK/TOR), cAMP/protein kinase A (cAMP/PKA) and insulin/insulin-like growth factor 1 (IGF-1) signaling pathways and also incorporates a sirtuin-governed protein deacetylation module [4-14]; 2) a cytoprotective process of autophagy [8-9,15-23]; and 3) tricarboxylic acid cycle, respiration, and reactive oxygen species (ROS) production and detoxification in mitochondria [24-32]. Second, some of the proteins implemented in such convergent mechanisms could function as oncoproteins, whereas others act as tumor suppressor proteins [3-6,10-21,24-33]. Third, certain pharmacological and dietary interventions exhibit both anti-aging and anti-cancer effects by modulating the longevity signaling network that integrates the AMPK/TOR, cAMP/PKA and insulin/IGF-1 pathways and incorporates the sirtuin-governed protein deacetylation module [4-7,10-14,33-52].

The interplay between aging and cancer is more complex than only sharing common aetiology and having convergent mechanisms. In some situations aging and cancer can have antagonistic aetiologies and divergent mechanisms [5,8,9,53-65]. Indeed, the age-dependent accumulation of DNA damage and mutations in normal somatic cells (especially in adult stem and progenitor cells) triggers telomere shortening and/or a gradual rise in the expression of the *INK4a/ARF* locus encoding the p16INK4a and p14ARF/p19ARF tumor suppressor proteins [53-56,58,62]. Both these processes reduce the proliferative potential of normal somatic cells, thereby promoting cellular senescence, causing a decline in tissue regeneration and repair, impairing tissue homeostasis, and ultimately accelerating cellular and organismal aging [53-58]. While both telomere shortening and enhanced expression of *INK4a/ARF* display pro-aging effects in normal somatic cells, they exhibit potent anti-cancer effects in tumor cells by reducing their proliferative potential [53-62]. Hence, an anti-cancer intervention that can limit the excessive proliferation of tumor cells by inhibiting telomerase or activating expression of *INK4a/ARF* could have a pro-aging effect on cellular and organismal levels [5,8,9,55-59,62-65].

The complexity of the interplay between aging and cancer is further underscored by the recent findings implying that tumor cells in the epithelia of breast cancers can cause “accelerated aging” of adjacent normal fibroblasts by stimulating their intracellular ROS production [66-77]. In response to the resulting oxidative stress these fibroblasts establish a pro-aging pattern by activating aerobic glycolysis and autophagic degradation, thereby providing epithelial cancer cells within the tumor microenvironment with lactate, ketone bodies and glutamine [67,70,76-79]. These catabolic and anabolic substrates support proliferation of epithelial cancer cells and, thus, accelerate tumor growth, progression and metastasis [67,76,77]. Further emphasizing the complexity of the relationship between aging and cancer, this model of breast cancer as an “accelerated host aging” disease defines autophagy (a cytoprotective anti-aging cellular process [8,9,15-23]) within cancer-associated fibroblasts as a pro-cancer process that supports the growth of already established tumors [67,76,77]. In contrast, by preventing initiation of some cancers, autophagy operates as an anti-cancer process prior to tumor establishment [8,9,15-23,80-85].

We found that lithocholic acid (LCA), a bile acid, delays chronological aging of yeast [86] known to mimic aging of postmitotic mammalian cells (*e.g.*, neurons) [87-89]. In yeast, LCA extends longevity not by attenuating the pro-aging AMPK/TOR and cAMP/PKA signaling pathways [86], both of which operate as pro-cancer pathways in mice and humans [4-14]. Furthermore, we revealed that the longevity-extending effect of LCA in chronologically aging yeast depends on autophagy (Kyryakov et al., manuscript in preparation), a cytoprotective anti-aging process that in different situations can play either an anti-cancer or a pro-cancer role [8,9,15-23,80-85]. Moreover, LCA extends yeast chronological life span by altering the age-related dynamics of mitochondria-confined respiration and ROS production [86], both of which are integrated into convergent mechanisms underlying aging and cancer [24-32]. Additionally, we found that in chronologically aging yeast LCA slows down telomere shortening (Iouk et al., unpublished data), a pro-aging process that exhibits an anti-cancer effect [53-62]. Besides, we demonstrated that LCA not only extends the chronological life span of quiescent yeast but also hinders cellular quiescence and senescence by delaying replicative aging of yeast (Lindsay et al., manuscript in preparation), which mimics aging of mitotically active mammalian cells [87-89]. In sum, these findings imply that in yeast models of aging of mitotically active and inactive mammalian cells LCA extends longevity by modulating several processes integrated into either convergent or divergent mechanisms underlying aging and cancer.

We therefore sought to examine if LCA exhibits an anti-tumor effect in cultured human cancer cells by activating certain anti-cancer processes that may play an essential role in cellular aging. As a model for assessing such effect of LCA, we choose several cell lines of the human neuroblastoma (NB) tumor. NB is the most commonly diagnosed extra-cranial solid tumor among children [90-92]. All NBs originate from primordial neuroblast cells that eventually differentiate into the adrenal medulla and early sympathetic nervous system [92-95]. In over 70% of all cases studied so far, NBs metastasize to other tissues [90-95]. Although most patients diagnosed with non-metastasizing NBs have been reported to be cured, only less than 40% of those with cancerous migration to other tissues survive despite rigorous chemotherapeutic and surgical treatment [92]. The recurrent high-risk NB patients with an often fatal prognosis frequently contain the following two genetic abnormalities: 1) amplification of the MYCN gene [96-98] encoding a transcription factor involved in growth, cell metabolism and division [99]; and 2) deletion of the short arm of chromosome 1p [100], which reduces expression of numerous tumour-suppressing genes [101-104]. One of the main challenges of combating high-risk recurring NBs is the use of non-toxic agents that not only prevent tumor metastasis, but also eliminate the primary tumor. Currently, NBs are treated with a combination of the chemotherapeutic drugs that cause apoptotic death of malignant cancer cells by inducing DNA strand intercalation (doxorubicin, cisplatin, cyclophosphamide and topotecan), DNA strand breaks (epipodophyllotoxins) or mitotic inhibition (vincristine) [105-111]. Noteworthy, all these anti-NB drugs stimulate the release of significant amount of ROS from mitochondria [107-111].

Here we show that LCA has a potent anti-tumor effect in four lines of cultured human NB cells. We demonstrate that this anti-aging compound kills three of them by causing apoptotic cell death. Our mechanistic studies of the apoptosis-based death of NB cell cultures exposed to LCA provide evidence that this bile acid triggers both the intrinsic and extrinsic apoptotic death pathways by binding to the cell surface and initiating the converging intracellular cascades activating caspases-3, -6, -8 and -9. We propose a model for a mechanism underlying a potent and specific anti-tumor effect of LCA in cultured human NB cells. We also show that LCA has a broad anti-tumor effect on cultured cancer cells derived from different tissues and organisms.

## Results

### LCA selectively kills cultured human NB cells

**Figure 1:**
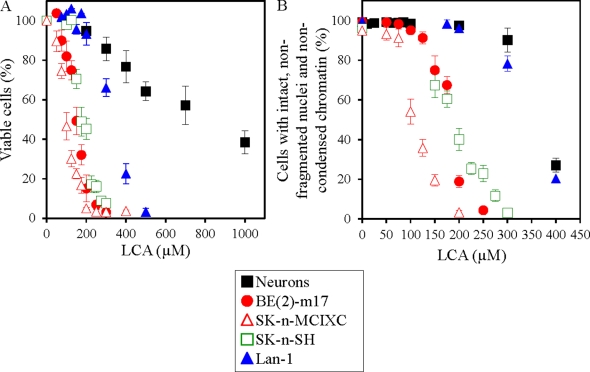
LCA selectively kills all four tested lines of cultured human NB cells - by causing an apoptosis-based death of BE(2)-m17, SK-n-SH and SK-n-MCIXC lines, but triggering a non-apoptotic death of Lan-1 line In A, the percentage of viable cells was calculated as a portion of their population displaying a detectable level of redox potential which was monitored using the MTT (3-(4,5-dimethylthiozol-2-yl)-2,5-diphenyltetrazolium bromide)-based CellTiter 96 Non-Radioactive Cell Proliferation Assay. In B, the fluorescent dye Hoechst was used to visualize chromatin in various cell cultures and the percentage of viable non-apoptotic cells carrying intact, non-fragmented nuclei containing non-condensed chromatin was calculated; dead apoptotic cells carried fragmented nuclei containing condensed chromatin, a hallmark event of apoptotic death. A. LCA kills NB cell lines BE(2)-m17, SK-n-SH, SK-n-MCIXC and Lan-1 if used at concentrations that are not cytotoxic or only mildly cytotoxic (as in case of Lan-1) to primary cultures of human neurons. B. The NB cell line SK-n-MCIXC is the most sensitive to LCA-induced apoptotic death; two other lines, BE(2)-m17 and SK-n-SH, exhibit much higher sensitivity to LCA-induced apoptotic death than primary cultures of human neurons or the NB cell line Lan-1. Data are presented as means ± SD (n = 4-6).

The MTT (3-(4,5-dimethylthiozol-2-yl)-2,5-diphenyltetrazolium bromide) assay is widely used to determine viability of cultured mammalian cells following drug treatment [112-116]. In this assay, only viable, metabolically active cells are able to reduce the yellow tetrazolium salt of MTT to form a purple formazan product. Thus, the percentage of viable cells in the MTT assay is calculated as the proportion of the cell population displaying a detectable level of redox potential. Using this assay, we found that LCA exhibits a potent and selective anti-tumor effect in several lines of cultured human NB cells. In fact, this bile acid killed the NB cell lines BE(2)-m17, SK-n-SH, SK-n-MCIXC and Lan-1 if used at concentrations that were not cytotoxic or only mildly cytotoxic (as in case of Lan-1) to primary cultures of human neurons (Fig. 1A).

Chromatin condensation and nuclear fragmentation are hallmark events of apoptosis that are not seen under any other modes of cell death [117-122]. We used the fluorescent dye Hoechst to visualize chromatin in various NB cell cultures and in primary cultures of human neurons exposed to LCA or remained untreated. For each cell culture, we calculated the percentage of viable non-apoptotic cells carrying intact, non-fragmented nuclei containing non-condensed chromatin. Dead apoptotic cells carried condensed and/or fragmented nuclei. Our data imply that if LCA is used at concentrations that are not cytotoxic to primary cultures of human neurons, it selectively kills the NB cell lines BE(2)-m17, SK-n-SH and SK-n-MCIXC by causing their apoptotic death. We found that SK-n-MCIXC is the most sensitive (as compared to other NB cell lines tested and especially as compared to primary cultures of human neurons) to the apoptotic death induced by LCA (Fig. 1B), as it is to the MTT-monitored cytotoxic effect of this bile acid (Fig. 1A). Furthermore, although BE(2)-m17 and SK-n-SH were less sensitive to LCA-induced apoptosis than SK-n-MCIXC, they exhibited much higher sensitivity to such form of cell death (Fig. 1B) - and to the MTT-monitored cytotoxic effect of LCA (Fig. 1A) - than primary cultures of human neurons or the NB cell line Lan-1. Importantly, although at the concentrations of LCA exceeding 200 μM the NB cell line Lan-1 was more sensitive to the MTT-monitored cytotoxic effect of LCA than primary cultures of human neurons (Fig. 1A), this cell line did not exhibit higher sensitivity to LCA-induced apoptotic cell death than primary neuron cultures (Fig. 1B). Thus, LCA selectively kills Lan-1 cells via a non-apoptotic cell death mechanism.

### LCA sensitizes two NB cell lines to hydrogen peroxide-induced apoptotic cell death

**Figure 2:**
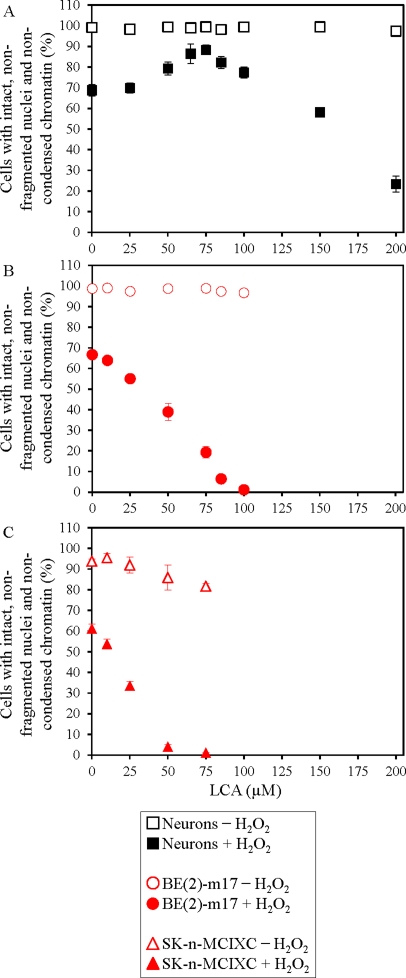
LCA increases sensitivity of cultured human NB cells to hydrogen peroxide-induced apoptotic cell death A. If used at concentrations below 100 μM, LCA protects primary cultures of human neurons against mitochondria-controlled apoptosis induced in response to exogenously added 0.1 mM hydrogen peroxide. B and C. In contrast, if used at concentrations below 100 μM, LCA greatly enhances the susceptibility of cultured human NB cell lines BE(2)-m17 (B) and SK-n-MCIXC (C) to such hydrogen peroxide-induced form of cell death. While 75 μM LCA causes apoptotic death of all or most of the cells of BE(2)-m17 and SK-n-MCIXC exposed to 0.1 mM hydrogen peroxide (B and C, respectively), in this concentration it increases the resistance of primary cultures of human neurons to such form of cell death (A). Nuclear fragmentation and chromatin condensation were visualized with the fluorescent dye Hoechst and were used as a measure of the efficacy of hydrogen peroxide-induced apoptotic cell death. Each panel shows two datasets for the same cell type. One dataset is for cell aliquots treated with 0.1 mM hydrogen peroxide, whereas the other dataset is for cell aliquots remained untreated. For each cell culture, the percentage of viable non-apoptotic cells carrying intact, non-fragmented nuclei containing non-condensed chromatin was calculated. Data are presented as means ± SD (n = 3-5).

Exogenously added hydrogen peroxide has been shown to cause mitochondria-controlled apoptotic cell death in a variety of cultured eukaryotic cells [123-128]. Our data imply that LCA protects primary cultures of human neurons, but not cultured human NB cell lines BE(2)-m17 or SK-n-MCIXC, against apoptosis induced in response to exogenously added 0.1 mM hydrogen peroxide (Fig. 2). In these experiments, chromatin condensation and nuclear fragmentation were visualized with the fluorescent dye Hoechst and were used as a measure of the efficacy of hydrogen peroxide-induced apoptotic cell death. Importantly, LCA greatly enhanced the susceptibility of BE(2)-m17 and SK-n-MCIXC to such a mitochondria-controlled form of cell death (compare Figs. 2B and 2C to Fig. 2A), even if it was used at concentrations that in the absence of hydrogen peroxide did not compromise their viability (compare Figs. 2B and 2C to Figs. 1A and 1B). Of note, while 75 μM LCA caused apoptotic death of all or most of the BE(2)-m17 and SK-n-MCIXC cells exposed to hydrogen peroxide, this concentration of LCA significantly increased the resistance of primary cultures of human neurons to the hydrogen peroxide-induced form of mitochondria-controlled apoptotic death (compare Fig. 2A to Figs. 2B and 2C). Based on these observations, it seems likely that an exposure of a mixed population of at least these two NB cell lines and non-cancerous neurons to low concentrations of simultaneously added hydrogen peroxide and LCA may concurrently 1) kill all or most of NB cells by causing their apoptotic cell death; and 2) promote the viability of non-cancerous neurons by increasing their resistance to hydrogen peroxide-induced apoptosis.

### LCA triggers the intrinsic (mitochondrial) pathway of apoptotic death in two NB cell lines

**Figure 3:**
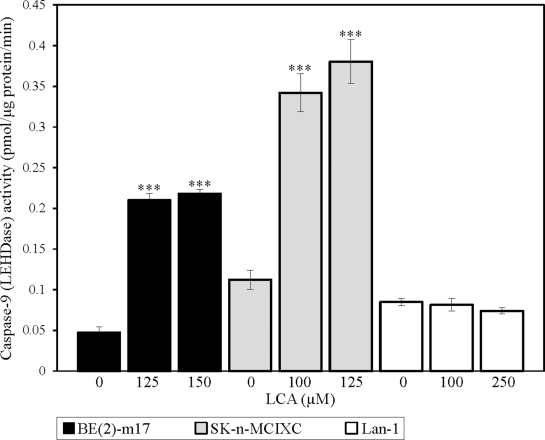
LCA significantly increases the activity of the initiator caspase-9 in cultured human NB cell lines BE(2)-m17 and SK-n-MCIXC Specific activity of caspase-9 was measured as described in Materials and Methods and expressed in picomoles of fluorescent compound 7-amino-4-trifluoromethyl coumarin released per microgram of protein per minute, based on the linear range of the curve. Data are presented as means ± SD (n = 3-4); ***p<0.001.

**Figure 4:**
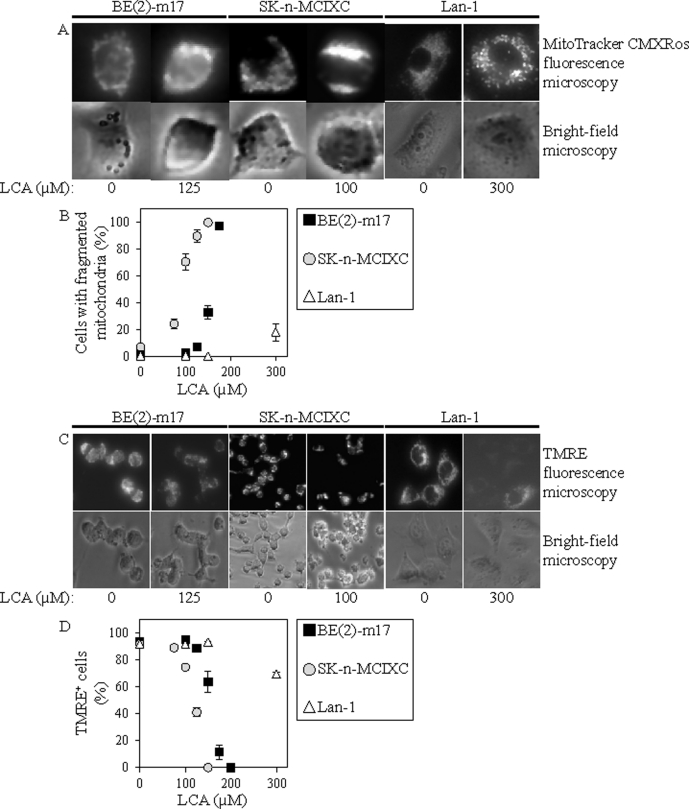
In cultured human NB cell lines BE(2)-m17 and SK-n-MCIXC (but not in Lan-1), LCA causes mitochondrial fragmentation and triggers the dissipation of the electrochemical potential across the inner mitochondrial membrane A. Mitochondrial morphology in NB cells treated with LCA or remained untreated was visualized using MitoTracker Red CMXRos as described in Materials and Methods. Cells were viewed using fluorescence microscopy. B. The percentage of cells displaying fragmented mitochondria was calculated. C. The mitochondrial membrane potential (∆Ψ) in NB cells treated with LCA or remained untreated was measured using tetramethylrhodamine ethyl ester (TMRE) as described in Materials and Methods; the extent of reversible sequestration of TMRE by mitochondria is proportional to the value of ∆Ψ. Cells were incubated with 50 nM TMRE for 20 min and directly viewed using fluorescence microscopy. D. The percentage of TMRE-positive cells displaying a detectable level of ∆Ψ was calculated. Data in B and D are presented as means ± SD (n = 3-4).

As described above, LCA selectively kills the NB cell lines BE(2)-m17 and SK-n-MCIXC by causing apoptosis and enhances their susceptibility to a hydrogen peroxide-induced form of mitochondria-controlled apoptosis. We therefore sought to investigate if LCA kills these two NB cell lines by activating the intrinsic apoptotic cell death pathway. The key feature of such a pathway is mitochondrial outer membrane permeabilization (MOMP), which leads to the release of cytochrome c (and several other pro-apoptotic proteins) from the mitochondrial intermembrane space into the cytosol [129-131]. Following its efflux from mitochondria, cytochrome c binds an apoptotic protease-activating factor 1 (APAF-1) monomer whose oligomerization into the heptameric apoptosome complex recruits caspase-9 and ultimately activates this initiator caspase [119,131-133]. We found that LCA significantly increases caspase-9 activity in BE(2)-m17 and SK-n-MCIXC (Fig. 3). Furthermore, in both these NB cell lines LCA caused the fragmentation of a mitochondrial network (Figs. 4A and 4B). Such fragmentation is one of the earliest events of the mitochondrial pathway of apoptosis; it occurs before caspase-9 activation, around the point of MOMP and cytochrome c efflux from mitochondria [119,129-131,134,135]. Moreover, in both these cell lines LCA triggered the dissipation of the electrochemical potential across the inner mitochondrial membrane (Figs. 4C and 4D). The gradual dissipation and eventual loss of mitochondrial transmembrane potential is a hallmark event of mitochondria-dependent apoptotic cell death; it follows MOMP and occurs in both caspase-dependent and caspase-independent fashion [119,130,131,136-139]. Of note, the higher LCA-induced activity of caspase-9 (Fig. 3) and efficacies of both LCA-promoted mitochondrial fragmentation and LCA-triggered mitochondrial inner membrane depolarization (Figs. 4B and 4D) observed in SK-n-MCIXC cells, as compared to those seen in BE(2)-m17 cells, correlated with the higher sensitivity of SK-n-MCIXC (as compared to that of BE(2)-m17) to the cytotoxic, apoptotic cell death-inducing and hydrogen peroxide-sensitizing effects of LCA (Figs. 1 and 2).

Importantly, although LCA stimulated caspase-9 in BE(2)-m17 and SK-n-MCIXC cells, it did not increase caspase-9 activity in Lan-1 cells (Fig. 3). Furthermore, if used at a concentration (*i.e.*, 150 μM or 175 μM) that caused mitochondrial fragmentation in most or all BE(2)-m17 and SK-n-MCIXC cells, LCA did not stimulate such fragmentation in Lan-1 cells (Fig. 4B). Moreover, if used at a concentration (*i.e.*, 150 μM or 200 μM) that triggered mitochondrial inner membrane depolarization in most or all BE(2)-m17 and SK-n-MCIXC cells, LCA did not cause such depolarization in Lan-1 cells (Fig. 4D). These findings further validate our conclusion (see above) that LCA kills Lan-1 cells via a non-apoptotic cell death mechanism.

### LCA increases activities of caspases-3 and -6 in two NB cell lines

The apoptosome-driven activation of caspase-9 during mitochondria-controlled apoptosis initiates a stepwise activation of several executioner caspases. In one scenario, the caspase-9-dependent proteolytic activation of the executioner caspases-3 and -7 is followed by the caspase-3-driven proteolytic activation of the executioner caspases-2 and -6 and then by the caspase-6-enabled proteolytic activation of the executioner caspase-10; when all these caspases become activated, they complete the demolition phase of the mitochondria-controlled apoptotic program by cleaving their respective protein substrates and producing the morphological features characteristic of apoptotic cell death [119,130,131,140,141]. In another scenario, caspase-6 is activated independent of caspase-3 [142-151], either by caspase-1 [148] or through intramolecular self-cleavage [152-154], and does not induce apoptosis [143,144,149,152,155].

**Figure 5:**
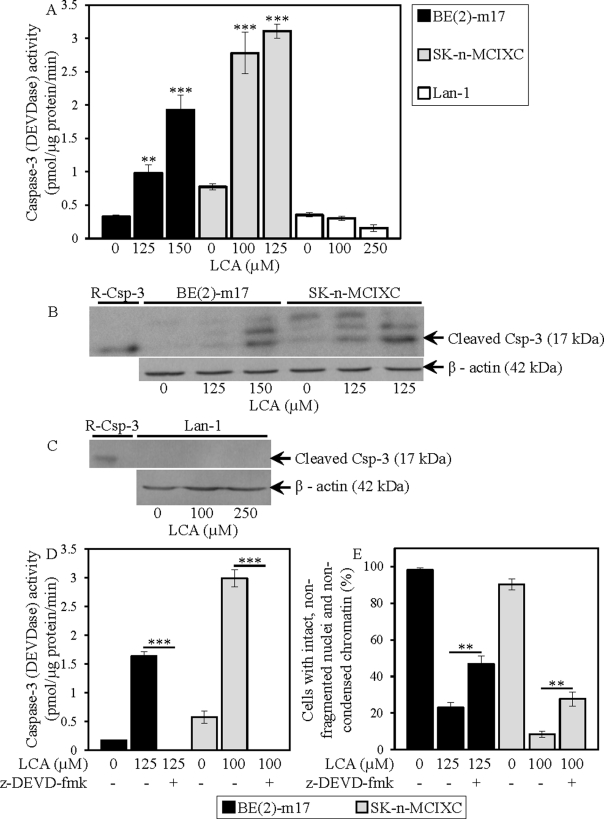
LCA increases activity of the executioner caspase-3 in cultured human NB cell lines BE(2)-m17 and SK-n-MCIXC (but not in Lan-1) by stimulating a proteolytic conversion of its zymogen pro-caspase-3 form into its enzymatically active 17 kDa form The anti-tumor effect of LCA in BE(2)-m17 and SK-n-MCIXC cell lines is due in part to its ability to increase caspase-3 activity. A and D. Specific activity of caspase-3 was measured in NB cells treated with LCA and/or z-DEVD-fmk (a potent and specific inhibitor of caspase-3) or remained untreated as described in Materials and Methods and expressed in picomoles of fluorescent compound 7-amino-4-trifluoromethyl coumarin released per microgram of protein per minute. B and C. Pro-caspase-3 cleavage assay was carried out as described in Materials and Methods. E. The fluorescent dye Hoechst was used to visualize chromatin in NB cell cultures treated with LCA and/or z-DEVD-fmk or remained untreated. For each cell culture, the percentage of viable non-apoptotic cells carrying intact, non-fragmented nuclei containing non-condensed chromatin was calculated; dead apoptotic cells carried fragmented nuclei containing condensed chromatin, a hallmark event of apoptotic death. Data in A, D and E are presented as means ± SD (n = 3-4); **p<0.01; ***p<0.001.

**Figure 6:**
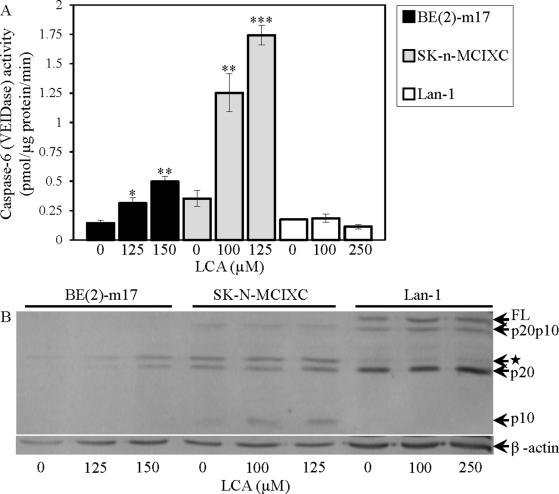
LCA increases activity of the executioner caspase-6 in cultured human NB cell lines BE(2)-m17 and SK-n-MCIXC (but not in Lan-1) by stimulating a proteolytic conversion of a zymogen pro-caspase-6 form into its large and small subunits known to assemble into an enzymatically active heterotetramer consisted of the two of each subunits A. Specific activity of caspase-6 was measured as described in Materials and Methods and expressed in picomoles of fluorescent compound 7-amino-4-trifluoromethyl coumarin released per microgram of protein per minute. B. Pro-caspase-6 cleavage assay was carried out as described in Materials and Methods. The anti-human-caspase-6 mouse monoclonal IgG1 clone B93-4 antibodies raised against amino acids 271-285 (Pharmingen) were used; these antibodies specifically recognize the large (p20) and small (p10) subunits of an enzymatically active form of caspase-6. Abbreviations: FL, a full-length zymogen form of pro-caspase-6; p20p10, caspase-6 lacking the pro-domain; p20 and p10, large and small subunits (respectively) of caspase-6 lacking the pro- and linker-domains; ★, an unidentified protein band, perhaps p20-linker (p23), pro-p20 (p23) or pro-p20-linker subunits of caspase-6 [152]. Data in A are presented as means ± SD (n = 3-4); *p<0.05; **p<0.01; ***p<0.001.

We found that LCA increases caspase-3 activity in BE(2)-m17 and SK-n-MCIXC (Fig. 5A) by promoting a proteolytic conversion of a zymogen pro-caspase-3 form into an active 17 kDa form (Fig. 5B). Importantly, the anti-tumor effect of LCA in these two cell lines was due in part to its ability to activate caspase-3. In fact, z-DEVD-fmk, a potent and specific inhibitor of caspase-3 (Fig. 5D), significantly reduced the efficacy with which LCA caused selective killing of BE(2)-m17 and SK-n-MCIXC cells (Fig. 5E). The observed incomplete reduction by z-DEVD-fmk of the anti-tumor efficacy of LCA in BE(2)-m17 and SK-n-MCIXC (Fig. 5E) suggests that the LCA-triggered, mitochondria-driven apoptotic death of both these cell lines could be due not only to a caspase-dependent mechanism, but also to a caspase-independent decline in mitochondrial function, which may lead to the observed LCA-dependent mitochondrial fragmentation and loss of mitochondrial transmembrane potential (Fig. 4). In BE(2)-m17 and SK-n-MCIXC cells, LCA also elevated caspase-6 activity (Fig. 6A) and promoted a proteolytic conversion of a zymogen pro-caspase-6 form of this caspase into its large (p20) and small (p10) subunits (Fig. 6B) known to assemble into an enzymatically active heterotetramer of two p20 and two p10 [152,156]. Of note, the different efficacies of the LCA-induced proteolytic activation of caspase-3 and caspase-6 seen in BE(2)-m17 and SK-n-MCIXC cells (Figs. 5 and 6) correlated with their dissimilar sensitivities to the cytotoxic, apoptosis-inducing and hydrogen peroxide-sensitizing effects of LCA (Figs. 1 and 2). Furthermore, even if used at a higher concentration (*i.e.*, 250 μM) than the ones causing the highest extent of the LCA-induced proteolytic activation of caspase-3 and caspase-6 (*i.e.*, 125 μM or 150 μM) in SK-n-MCIXC and BE(2)-m17 cells, LCA did not promote their proteolysis-driven activation in Lan-1 cells (Figs. 5 and 6), whose LCA-induced death was not due to apoptosis (see above).

### LCA increases the activity of the initiator caspase-8 in two NB cell lines

The proteolytic cleavage of pro-caspase-3 and the resulting increase of caspase-3 activity can be triggered not only through the intrinsic (mitochondrial) apoptotic death pathway but also through the extrinsic (death receptor) pathway of apoptosis. This extrinsic pathway is initiated when specific extracellular death ligands cause the ligation of death receptors in the plasma membrane, thus triggering a process eventually leading to activation of the initiator caspase-8 [119,131]. In addition to its role in the proteolytic activation of caspase-3, activated in response to death receptor ligation caspase-8 can also cleave and activate the BH3-only protein BID, thereby causing MOMP and initiating the intrinsic pathway of apoptosis [119,131,157,158].

**Figure 7:**
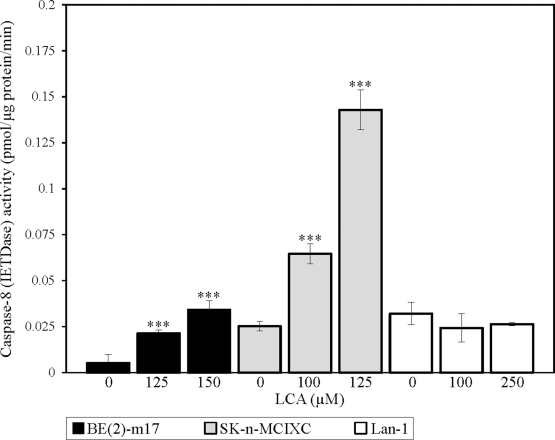
LCA increases activity of the initiator caspase-8 in cultured human NB cell lines BE(2)-m17 and SK-n-MCIXC (but not in Lan-1) Specific activity of caspase-8 was measured as described in Materials and Methods and expressed in picomoles of fluorescent compound 7-amino-4-trifluoromethyl coumarin released per microgram of protein per minute, based on the linear range of the curve. Data are presented as means ± SD (n = 3-4); ***p<0.001.

We found that LCA significantly increases the activity of caspase-8 in BE(2)-m17 and SK-n-MCIXC (Fig. 7). As it was observed for caspases-3, -6 and -9 (Figs. 5A, 6A and 3, respectively), the extent to which caspase-8 was activated by LCA in these two cell lines (Fig. 7) correlated with the degree of their sensitivity to the cytotoxic, apoptosis-inducing and hydrogen peroxide-sensitizing effects of LCA (Figs. 1 and 2). Importantly, unlike the stimulating effect of LCA on caspase-8 seen in BE(2)-m17 and SK-n-MCIXC cells, this bile acid did not stimulate caspase-8 activity in Lan-1 cells (Fig. 7). These findings suggest that LCA could kill both BE(2)-m17 and SK-n-MCIXC cell lines by activating not only the intrinsic apoptotic death pathway dependent on the initiator caspase-9, but also the extrinsic pathway of apoptotic death driven by the initiator caspase-8. We therefore propose that the LCA-dependent activation of caspase-8 seen in BE(2)-m17 and SK-n-MCIXC cells (Fig. 7) could be responsible not only for the direct proteolytic activation of caspase-3 (Figs. 5A and 5B), but also for MOMP (perhaps, due to BID cleavage by caspase-8) and the resulting initiation of the intrinsic apoptotic death pathway observed in these NB cells (Figs. 3 and 4). These caspase-8-driven events in BE(2)-m17 and SK-n-MCIXC cells are expected to enhance the extent of their demise in response to LCA treatment.

### LCA reduces the activity of the inflammatory caspase-1 in two NB cell lines

In addition to its stimulatory effect on caspases-3, -6, -8 and -9, LCA significantly reduced caspase-1 activity in NB cell lines BE(2)-m17 and SK-n-MCIXC, but not in Lan-1 (Suppl. Fig. 1). Caspase-1 is an inflammatory caspase that drives the processing and unconventional secretion of the cytokines interleukin-1β and interleukin-18 [159-162]. Following their secretion by mammalian cells, cytokines promote the growth and proliferation of neighbouring cells in the same tissue [163-166]. Hence, it is tempting to speculate that by reducing caspase-1 activity in BE(2)-m17 and SK-n-MCIXC cells, and thereby impairing their ability to process and secrete cytokines, LCA could prevent growth and proliferation of neighbouring NB cells in culture.

### LCA does not enter cultured NB cells

Our mass spectrometry-based measurement of cellular and extracellular levels of exogenously added LCA revealed that it does not enter BE(2)-m17, SK-n-MCIXC or Lan-1 cells (Suppl. Table 1). Thus, LCA selectively kills these cancer cells by binding to their surface and then initiating intracellular signaling cascades that cause their apoptotic (as in case of BE(2)-m17 and SK-n-MCIXC) or non-apoptotic (as in case of Lan-1) death. In BE(2)-m17 and SK-n-MCIXC cells, the intracellular signaling cascades triggered by LCA bound to their surface also reduce the activity of the inflammatory caspase-1.

### LCA kills cultured human breast cancer (BC) and rat glioma (GL) cells

In addition to its high anti-tumor efficacy and specificity in several NB cell lines, LCA displayed an anti-tumor effect in two other types of cultured cancer cells. In fact, treatment of the drug-sensitive human BC cell line MCF7 with LCA demonstrated a dose-dependent killing effect, as measured by the MTT cell viability assay (Suppl. Fig. 2A). Treatment with 1 μg/ml doxorubicin demonstrated that these BC cells were highly drug sensitive (Suppl. Fig. 2A). Importantly, killing of MCF7 cells with LCA was associated with DNA damage, as visualized with antibodies against phosphorylated histone H2AX (Suppl. Fig. 2B). Once cells encounter DNA damage, histone H2AX phosphorylation at sites of damage has been shown to initiate the chromatin response required for DNA repair [167]. This is believed to serve as a docking site for DNA repair enzymes. At doses as low as 25 and 50 μM LCA, increased phosphorylated H2AX could be visualized in MCF7 cells (Suppl. Fig. 2B). Since most of these BC cells remain viable at 50 μM LCA (Suppl. Fig. 2A), DNA damage likely precedes their killing by LCA. We also assessed the ability of LCA to kill the cultured rat GL cell line F98. These highly proliferative and invasive cancer cells have increased resistance to chemo- and radiation therapy [168]. We found that at a concentration of 100 μM, LCA kills most F98 cells (Suppl. Fig. 3). Together, our findings imply that LCA has a broad anti-tumor effect on cultured cancer cells derived from different tissues and organisms.

## Discussion

### Mechanisms underlying an anti-tumor effect of LCA in human NB cell cultures

This study provides evidence that LCA, a bile acid that delays aging of quiescent and proliferating yeast, exhibits a potent and selective anti-tumor effect in cultured human NB cells. Our findings suggest a model for two mechanisms underlying such effect of LCA, as outlined below. Several aspects of this model remain hypothetical.

**Figure 8:**
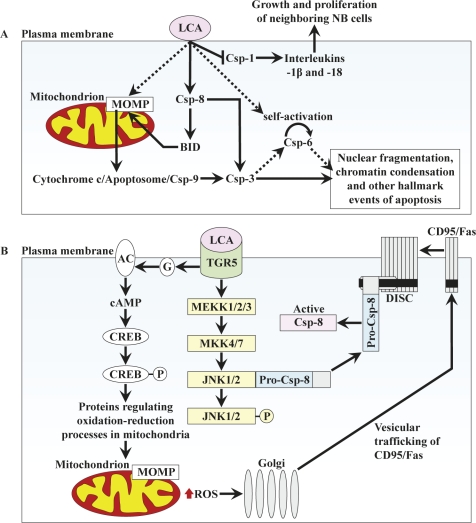
A model for a mechanism underlying an anti-tumor effect of LCA in cultured human NB cell lines BE(2)-m17 and SK-n-MCIXC See text for details. Abbreviations: AC, adenylyl cyclase; BID, BH3-interacting domain death agonist; CD95/Fas (factor-activating Exo S), death receptor; CREB, cAMP-response element binding protein; Csp-1, -3, -6, -8 and -9, caspases-1, -3, -6, -8 and -9; DISC, death-inducing signaling complex; G, a G-protein; JNK, c-Jun-N terminal kinase; LCA, lithocholic acid; MEKK1/2/3, mitogen-activated protein kinase/ERK kinase kinase-1, -2 and -3; MKK4/7, mitogen-activated protein kinase kinase-4 and -7; MOMP, mitochondrial outer membrane permeabilization; PKA, protein kinase A; ROS, reactive oxygen species; TGR5, a member of the G-protein coupled receptor family.

In NB cell lines BE(2)-m17 and SK-n-MCIXC, LCA triggers both the intrinsic and extrinsic pathways of apoptosis by binding to the cell surface and initiating the intracellular cascades that cause cell death (Fig. 8A). LCA binding to the cell surface leads to MOMP by activating caspase-8, which in turn causes MOMP - perhaps by cleaving and activating BID. LCA may also stimulate TGR5, its only known receptor on the cell surface (see below), which may then transmit a MOMP-activating signal from the plasma membrane to mitochondria via the cAMP/PKA signaling pathway. LCA-induced MOMP may result in efflux of cytochrome c from mitochondria, thereby causing apoptosome formation and the observed caspase-9 activation. Caspase-9 then activates caspase-3 by proteolytically processing pro-caspase-3. The proteolytic activation of caspase-3 is also carried out by caspase-8 operating in the LCA-induced extrinsic pathway of apoptosis. Active caspase-3 completes the demolition phase of the LCA-driven apoptotic program by cleaving its protein substrates. Furthermore, LCA binding to the cell surface leads to proteolytic activation of caspase-6; the mechanism underlying such activation (either self-cleavage or proteolytic processing by active caspase-3) and the involvement of caspase-6 in LCA-driven apoptosis remain to be established. Moreover, LCA binding to the cell surface causes inhibition of the inflammatory caspase-1. Such inhibition may contribute to the anti-tumor effect of LCA by attenuating the processing and secretion of the cytokines interleukin-1β and interleukin-18, thereby preventing growth and proliferation of neighbouring NB cells in culture.

Recent findings suggest the following cascade of events leading to the above described activation of both the intrinsic and extrinsic apoptotic pathways in response to LCA binding to the surface of BE(2)-m17 and SK-n-MCIXC cells. The only known LCA receptor on the cell surface in rodents and humans is TGR5, a member of the G-protein coupled receptor family [169,170]. LCA is the most potent natural agonist of TGR5, with EC_50_ of 530 nM [171,172]. Importantly, TGR5 has been shown to be expressed in neurons [170,173-175]. LCA-stimulated TGR5 could initiate three molecular cascades that trigger the intrinsic and extrinsic pathways of apoptotic cell death (Fig. 8B). First, it could initiate the cAMP/PKA signaling pathway known to response by altering oxidation-reduction processes in mitochondria and mitochondrial morphology [169,170,176-178]. The resulting changes in mitochondria may include such well-documented effects of LCA as MOMP activation and elevated ROS production [169,170,176-178]. Second, the elevated in response to LCA intracellular ROS could intensify vesicle-mediated trafficking of the CD95/Fas death receptor monomers from the Golgi to the plasma membrane, another well-documented effect of LCA in human cells [179,180]. Third, LCA-stimulated TGR5 could activate the MEKK1/2/3-MKK4/7 protein kinase cascade resulting in phosphorylation of c-Jun-N terminal kinase (JNK). Such LCA/TGR5-driven phosphorylation has been shown to release pro-caspase-8 from a complex that it forms with unphosphorylated JNK, thereby enabling its recruitment to the CD95/Fas death-inducing signaling complex consisting of the CD95/Fas death receptor trimers and the adaptor Fas-Associated Death Domain [176,181]. Following pro-caspase-8 dimerization and proteolytic processing, its active form could trigger both the intrinsic and extrinsic apoptotic death pathways (Fig. 8A).

Our findings imply that a different mechanism underlies the anti-tumor effect of LCA in cultured human NB cell line Lan-1. We propose such effect of LCA in Lan-1 is due to its ability to cause necrotic or some other non-apoptotic kind of death, which is not characterized by such major hallmark events of apoptosis as nuclear condensation and fragmentation, mitochondrial fragmentation and inner membrane depolarization, and activation of caspases-3, -6, -8 and -9.

A reason for the observed difference in the mechanisms by which LCA kills different NB cell lines (*i.e.*, by triggering apoptotic cell death of BE(2)-m17 and SK-n-MCIXC, but by causing non-apoptotic cell death of Lan-1) remains to be established. All these cell lines exhibit amplified expression of the MYCN gene [96-98] and deletion of the short arm of chromosome 1p [100-104]. However, the level of an anti-apoptotic member of the B-cell lymphoma-2 (BCL-2) protein family containing four BCL-2 homology (BH) domains in BE(2)-m17 is significantly lower than that in Lan-1 [182]. The anti-apoptotic BCL-2 proteins prevent MOMP [183,184]. Therefore, it is conceivable that the lower efficacy of MOMP prevention in BE(2)-m17 makes this cell line more susceptible to LCA-induced apoptosis (which involves MOMP activation) than Lan-1. Furthermore, it is plausible that, due to the relatively high level of the anti-apoptotic BCL-2 protein with four BH domains in Lan-1, this cell line is more susceptible (as compared to BE(2)-m17) to a MOMP-independent kind of LCA-induced non-apoptotic cell death.

Another kind of dissimilarity between different NB cell lines consists in lower activity of caspase-1 in BE(2)-m17 and SK-n-MCIXC cells (as compared to that in Lan-1 cells) that have not been exposed to LCA; importantly, LCA further and significantly reduces the activity of this inflammatory caspase in BE(2)-m17 and SK-n-MCIXC, but not in Lan-1 (Suppl. Fig. 1). This observation suggests that certain pro-inflammatory alterations (including elevated activity of caspase-1) in NB cells may play an anti-apoptotic, cytoprotective role. It is conceivable therefore that the lower activities of caspase-1 in BE(2)-m17 and SK-n-MCIXC (especially following their exposure to LCA) make these NB cell lines more susceptible to LCA-induced apoptosis than Lan-1, in which a relatively high activity of caspase-1 is not reduced by LCA. Of note, recent findings in mice imply that certain mild pro-inflammatory alterations, including increased mitochondrial ROS and elevated expression of inflammatory cytokines, can extend animal longevity [185,186].

### LCA modulates mitochondrial structure and function to cause an anti-tumor effect in cultured human NB cells and an anti-aging effect in quiescent yeast

This study as well as our published [86] and recent unpublished data suggest that both an anti-tumor effect of LCA in cultured human NB cells and its longevity-extending effect in quiescent yeast are due to its ability to modulate mitochondria-confined processes playing essential roles in both cancer and aging. Interestingly, the effects of LCA on these mitochondrial processes seen in NB cell cultures are opposite of those observed in quiescent yeast and, for some processes, in primary cultures of human neurons. Indeed, while LCA enhances the susceptibility of NB cell lines BE(2)-m17 and SK-n-MC to mitochondria-controlled apoptotic cell death caused by exogenously added hydrogen peroxide (Fig. 2), it significantly increases the resistance of quiescent yeast [86] and primary cultures of human neurons (Fig. 2) to such a form of cell death. Furthermore, while LCA causes the fragmentation of a tubular mitochondrial network into individual mitochondria in BE(2)-m17 and SK-n-MC (Figs. 4A and 4B), it attenuates such mitochondrial fragmentation in quiescent yeast [86]. Moreover, LCA triggers the complete dissipation of the electrochemical potential across the inner mitochondrial membrane in BE(2)-m17 and SK-n-MC (Figs. 4C and 4D). In contrast, although exposure of yeast cells to LCA causes a reduction of such potential by 50% upon entry into a non-proliferative state [86], for a long time after becoming quiescent these cells maintain higher mitochondrial transmembrane potential than their quiescent counterparts that have not been exposed to LCA (Beach et al., manuscript in preparation). To the best of our knowledge, these findings provide the first example of a compound that can kill cancer cells and increase longevity of non-cancerous cells by causing quite opposite effects on the same kind of mitochondria-confined processes in these two different cell types. A key challenge for the future will be to understand why mitochondria in cancer and non-cancerous cells respond so differently to LCA exposure.

### A broad anti-tumor effect of LCA

This study demonstrates that, in addition to the high anti-tumor efficacy and specificity exhibited by LCA in several NB cell lines, this bile acid displays an anti-tumor effect in human BC and rat GL cells. Future studies will examine if mechanisms underlying such broad anti-tumor effect of LCA are similar in cultured cancer cells that originate from different tissues and organisms.

## MATERIALS AND METHODS

### Cell culture

Human NB cell lines were cultured in the following media: 1) SK-n-MCIXC and BE(2)-m17 cells in a 1:1 mixture of Dulbecco's Modified Eagle's Medium (DMEM) and Ham's F-12 Nutrient Mixture (Invitrogen/Gibco BRL, Carlsbad, CA) supplemented with 10% fetal bovine serum (FBS) (HyClone, Logan, UT); 2) SK-n-SH cells in Eagle's Minimum Essential Medium (EMEM) (Invitrogen/Gibco BRL, Carlsbad, CA) supplemented with 10% FBS; and 3) Lan-1 cells in DMEM (Invitrogen/Gibco BRL, Carlsbad, CA) supplemented with 10% bovine calf serum (BCS) (HyClone, Logan, UT). Human primary neurons were prepared from fetal cerebrum tissue and cultured as previously described [187], in accordance with the Canadian Institute of Health Research regulations and as approved by the McGill University Institutional Review Board. MCF7 human BC and F98 rat GL cell lines from American Type Culture Collection were cultured in a 1:1 mixture of DMEM and Ham's F-12 Nutrient Mixture (Sigma-Aldrich, St Louis, MI) supplemented with 10% FBS (Invitrogen/Gibco BRL, Carlsbad, CA) and 1× anti-mycotic/anti-biotic (Invitrogen/Gibco BRL, Carlsbad, CA). All cell lines were cultured in a humidified atmosphere (5% CO_2_) at 37 °C.

### Treatment of cells with LCA, hydrogen peroxide or z-DEVD-fmk

Stock solutions of LCA (Sigma-Aldrich, St Louis, MI) in varying concentrations were first made in 100% dimethyl sulfoxide (DMSO) (Sigma-Aldrich, St Louis, MI). For treatment of cultured human NB, human BC and rat GL cells, these stock solutions were then diluted to the indicated final concentration of LCA (the final concentration of DMSO was always kept at 1%) in either a 1:1 mixture of DMEM and Ham's F-12 Nutrient Mixture supplemented with 10% FBS (for SK-n-MCIXC and BE(2)-m17 cells), EMEM supplemented with 10% FBS (for SK-n-SH cells), DMEM supplemented with 10% BCS (for Lan-1 cells) or a 1:1 mixture of DMEM and Ham's F-12 Nutrient Mixture supplemented with 10% FBS and 1X anti-mycotic/anti-biotic (for MCF7 and F98 cells). For treatment of human primary neurons with LCA, stock solutions of LCA in varying concentrations made in 100% DMSO were diluted in EMEM containing 0.225% sodium bicarbonate, 1 mM sodium pyruvate, 2 mM L-glutamine, 0.1% dextrose (all from Life Technologies, Gaithersburg, MD) and 5% FBS (HyClone, Logan, UT) to the indicated final concentration of LCA (the final concentration of DMSO was always kept at 1%). For treatment of cultured cancer cells or primary neurons with LCA, they were incubated for 48 hours in the presence LCA at the indicated final concentrations; control cells were treated with an empty DMSO vehicle only. For cell treatment with hydrogen peroxide, its 30% stock solution (Fisher Scientific, Waltham, MA) was diluted in sterile H_2_O and added directly to the cell cultures after 24 h of their pre-treatment with LCA or an empty DMSO vehicle only; cells were then incubated for 24 h. In experiments involving cell treatment with z-DEVD-fmk, this caspase-3 inhibitor was added to a final concentration of 5 μM simultaneously with LCA or an empty DMSO vehicle only.

### Cell viability assays

The number of viable cells in cultures exposed to LCA was measured using the MTT (3-(4,5-dimethylthiozol-2-yl)-2,5-diphenyltetrazolium bromide)-based CellTiter 96 Non-Radioactive Cell Proliferation Assay (Promega, Madison, WI). In this assay, only viable, metabolically active cells were able to reduce the yellow tetrazolium salt of MTT to form a purple formazan product. This insoluble product was solubilized by the addition of a detergent. The resulting intracellular purple formazan was then detected spectrophotometrically using a 96-well plate reader at a wavelength of 570 nm. The signal was corrected to account for cellular debris using a wavelength of 630 nm. Chromatin in cells exposed to LCA and/or hydrogen peroxide and/or z-DEVD-fmk was visualized with the fluorescent dye Hoechst used at a final concentration of 4 μM in culture media and viewed using fluorescence microscopy. For each cell culture, the percentage of viable non-apoptotic cells carrying intact, non-fragmented nuclei containing non-condensed chromatin was calculated. Dead apoptotic cells carried fragmented nuclei containing condensed chromatin, a hallmark event of apoptotic death.

### Visualization of mitochondria and measurement of the mitochondrial membrane potential by fluorescence microscopy

Mitochondrial morphology of cells treated with LCA was visualized using MitoTracker Red CMXRos (Molecular Probes, San Diego, CA) used at a concentration of 125 nM in the culture media. Cells were viewed using fluorescence microscopy, and the percentage of cells displaying fragmented mitochondria was calculated. The mitochondrial membrane potential (∆Ψ) was measured using tetramethylrhodamine ethyl ester (TMRE), a cell-permeant, cationic fluorescent dye. The extent of reversible sequestration of TMRE by mitochondria is proportional to the value of ∆Ψ. Cells were incubated with 50 nM TMRE for 20 min and directly viewed using fluorescence microscopy. The percentage of TMRE-positive cells displaying a detectable level of ∆Ψ was calculated.

### Fluorescence microscopy

For all cells and fluorescent dyes, images were collected with a Nikon Eclipse (TE2000-U) inverted fluorescence microscope under 20 × magnification. A filter cube with an excitation wavelength of 330-380 nm was used to visualize Hoechst-stained cells. Cells stained with MitoTracker Red CMXRos or TMRE were visualized using filter cubes with excitations of 590-650 nm or 532-587 nm, respectively.

### Caspase activity assays

Proteins were extracted from harvested cells using an ice-cold lysis buffer containing 50 mM HEPES, 0.1% CHAPS, 0.1 mM EDTA and protease inhibitors (0.1μg/ml TLCK, 0.5 μg/ml leupeptin, 38 mg/ml AEBSF, 0.1 μg/ml pepstatin A). The cell lysate was centrifuged at 13,000 × g for 5 min at 4°C to remove cell debris and any detergent-insoluble proteins. The supernatant was collected and frozen at -80°C. Extracts were thawed and protein concentrations were quantified with the Pierce BCA protein assay (Thermo Scientific, Waltham, MA). The extracts were then assayed for caspase activities using fluorogenic substrates specific for caspase-1 (10 μM Ac-YVAD-AFC), caspase-3 (5 μM Ac-DEVD-AFC), caspase-6 (10 μM Ac-VEID-AFC), caspase-8 (10 μM Ac-IETD-AFC) or caspase-9 (10 μM Ac-LEHD-AFC) in caspase reaction buffer (20 mM PIPES, pH 7.2, 30 mM NaCl, 10 mM DTT, 1 mM EDTA, 0.1% CHAPS, 10% sucrose). The time-dependent release of the fluorescent compound 7-amino-4-trifluoromethyl coumarin (AFC) was monitored using a Bio-Rad Fluoromark fluorometer (Hercules, CA) at an excitation wavelength of 390 nm and an emission wavelength of 538 nm. Measurements were recorded at 2 min intervals for 1 h. A standard curve of AFC fluorescence was used to calculate AFC (in picomoles) released in the reactions. Specific activities were expressed as picomoles of AFC released per microgram of protein per minute, based on the linear range of the curve.

### Pro-caspase-3 and pro-caspase-6 cleavage assays

50 μg of protein from crude cellular extracts were resolved by SDS-PAGE in a 15% polyacrylamide gel and then transferred to a PVDF Immobilon-P membrane (Millipore, Bedford, MA). Following wash with 5% milk, blots were incubated with primary antibodies used at the following dilutions: 1:500 for anti-cleaved-caspase-3 p17 (Cell Signaling, Beverly, MA), 1:250 for anti-cleaved-caspase-6 p10 (Pharmingen, San Diego, CA) and 1:5,000 for anti-β-Actin (Sigma-Aldrich, St Louis, MI). Immunoreactivity was visualized with horseradish peroxidase-conjugated secondary antibodies (Amersham, Oakville, ON) that were used at dilutions 1:2,000 (for anti-rabbit) and 1:5,000 (for anti-mouse), respectively. Antigen-antibody complexes were detected by enhanced chemiluminescence using an ECL plus chemiluminescence Western blot detection reagents (Amersham Pharmacia Biotech, Piscataway, NJ).

### Histone H2AX phosphorylation assay

BC cells were removed from 100-mm tissue culture dishes using a rubber policeman and ice-cold phosphate-buffered saline (PBS). The cells were pelleted by centrifugation at 3,000 rpm at 4°C. The pellets were resuspended in ice-cold lysis buffer (20 mM Tris-HCl pH 7.5, 150 mM NaCl, 0.5 mM EDTA, 0.1 mM EGTA, 0,1% NP-40, 1× mammalian cell anti-protease cocktail (Sigma-Aldrich, St Louis, MI)). The cells were lysed using multiple freeze-thaw cycles followed by pulse sonication on ice and centrifugation at 3,000 rpm for 5 min at 4°C to remove cell debris. Western blot analysis of these protein lysates was performed as previously described [188]. Briefly, equivalent amounts of protein (assessed using Bio-Rad Protein Reagent; Bio-Rad Laboratories, Hercules, CA) were resolved on 10% SDS-PAGE gels. Following electrophoresis the proteins were trans-blotted onto nitrocellulose membranes (Pall-VWR International, Radnor, PA). The membranes were blocked overnight at 4° C on a gyratory plate with 5% molecular grade skim milk powder (Bio-Rad Laboratories, Hercules, CA) in PBS containing 0.1% Tween-20 (PBST). Primary and secondary antibody incubations and subsequent washes were carried out in the same buffer. The antibody to phosphorylated H2AX was obtained from Santa Cruz Biotechnology (Santa Cruz, CA). The primary antibody for GAPDH was purchased from Sigma-Aldrich (St Louis, MI). Secondary HRP antibodies were purchased from Bio-Rad Laboratories (Hercules, CA). Blots were immunoprobed overnight with primary antibodies used at a 1:1,000 dilution. Secondary HRP antibody was applied at room temperature on a gyratory plate at a 1:10,000 dilution for 30 min. Following multiple washes, an enhanced chemiluminescence detection system (DuPont-NEN; Boston, MA) was used to detect the target antigen/antibody complexes. Blots were then stripped at 50° C for 30 min in a Tris-buffered 20% SDS/1% 2-mercaptoethanol stripping solution, washed and reprobed with GAPDH antibody to verify protein loading equivalency.

### Mass spectrometric measurement of LCA

Mass spectrometry-based quantitative analysis of LCA was performed as previously reported [189]. In brief, lipids were extracted by a modified Bligh and Dyer method [189] from cells pelleted by centrifugation for 5 min at 16,000 × g at 4°C and from the supernatant of cultural medium. The extracted lipids were dried under nitrogen and resuspended in chloroform. Immediately prior to injection the extracted lipids were combined with a 2:1 methanol:chloroform mixture supplemented with 0.1% (v/v) ammonium hydroxide. The sample was injected directly into a Q-TOF 2 mass spectrometer (Waters, Milford, MA) using a nano-ESI spray source at 1 μl/min. Spectra were obtained in a negative-ion mode. The cone voltage was set to 30 v, the capillary voltage to 3.2 kv and the collision gas to 10 (arbitrary units). Acquired spectra were centroided using the Masslynx software then deconvoluted and deisotoped with Excel macros.

### Statistical analysis

Statistical analysis was performed using Microsoft Excel's (2010) Analysis ToolPack-VBA. All data are presented as the means ± SD. The *p* values were calculated using an unpaired two-tailed *t* test.

## Supplementary Figures and Table


