# Supplementary material for: Lithocholic bile acid selectively kills neuroblastoma cells, while sparing normal neuronal cells
Source: Oncotarget. 2011 Oct 11;2(10):761–82. doi: 10.18632/oncotarget.338 (PMC3248158; doi:10.18632/oncotarget.338)
Supplement: Supplementary file 1 [file oncotarget-02-761-s001.pdf]

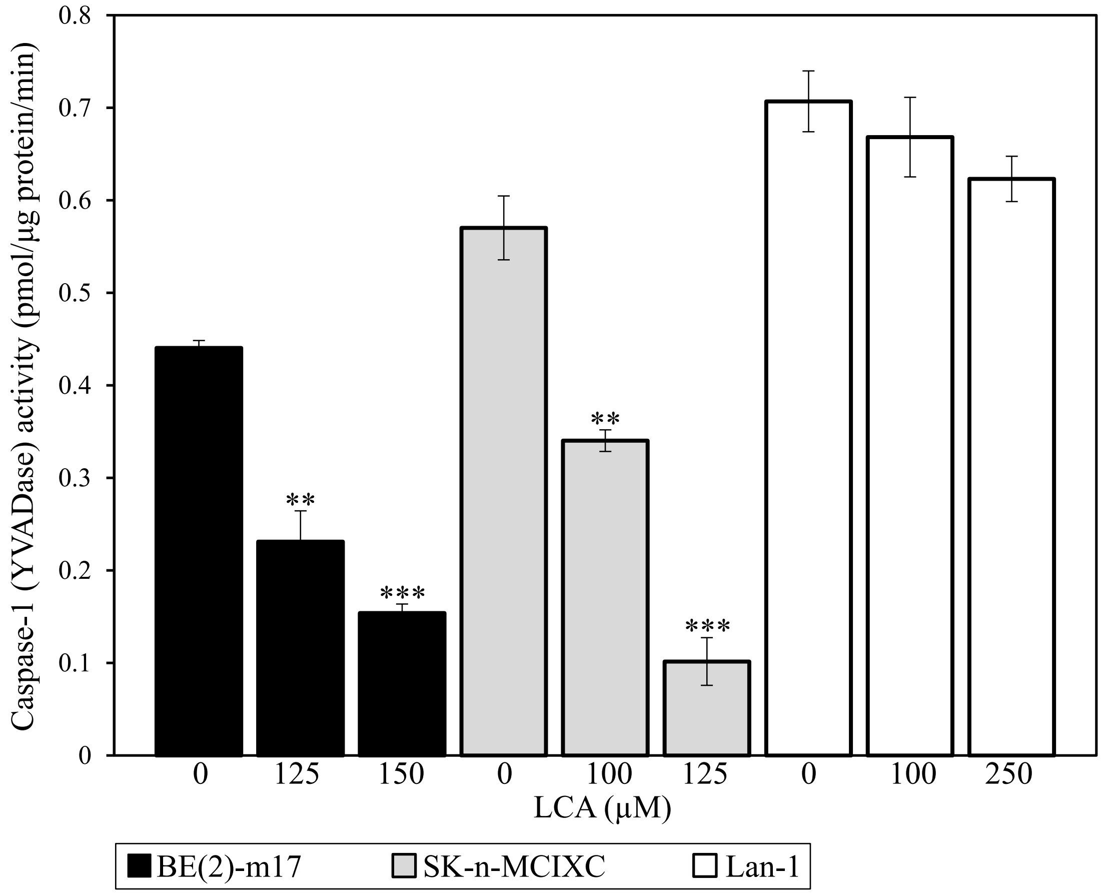

Supplemental Figure 1. LCA significantly reduces the activity of the inflammatory caspase-1 in cultured human NB cell lines BE(2)-m17 and SK-n-MCIXC, but not in Lan-1. Specific activity of caspase-1 was measured as described in Materials and Methods and expressed in picomoles of fluorescent compound 7-amino-4-trifluoromethyl coumarin released per microgram of protein per minute, based on the linear range of the curve. Data are presented as means ± SD (n = 3-4); *p<0.05; **p<0.01; ***p<0.001.

Cell line	LCA (μM) added	% of LCA recovered	
		Medium	Cells
BE(2)-m17	50	99.88 ± 0.02	0.12 ± 0.02
	75	99.92 ± 0.04	0.08 ± 0.04
	100	99.95 ± 0.03	0.05 ± 0.03
	125	99.98 ± 0.02	0.02 ± 0.02
SK-n-MCIXC	25	99.93 ± 0.05	0.07 ± 0.05
	50	99.88 ± 0.06	0.12 ± 0.06
	75	99.97 ± 0.03	0.03 ± 0.03
	100	99.95 ± 0.04	0.05 ± 0.04
Lan-1	100	99.91 ± 0.04	0.09 ± 0.04
	250	99.95 ± 0.05	0.05 ± 0.05

Supplemental Table 1. Exogenously added LCA does not enter cultured human NB cell lines BE(2)-m17, SK-n-MCIXC and Lan-1. LCA was extracted from cells and cultural media and then measured using mass spectrometry, as described in Materials and Methods. Data are presented as means ± SD (n = 3).

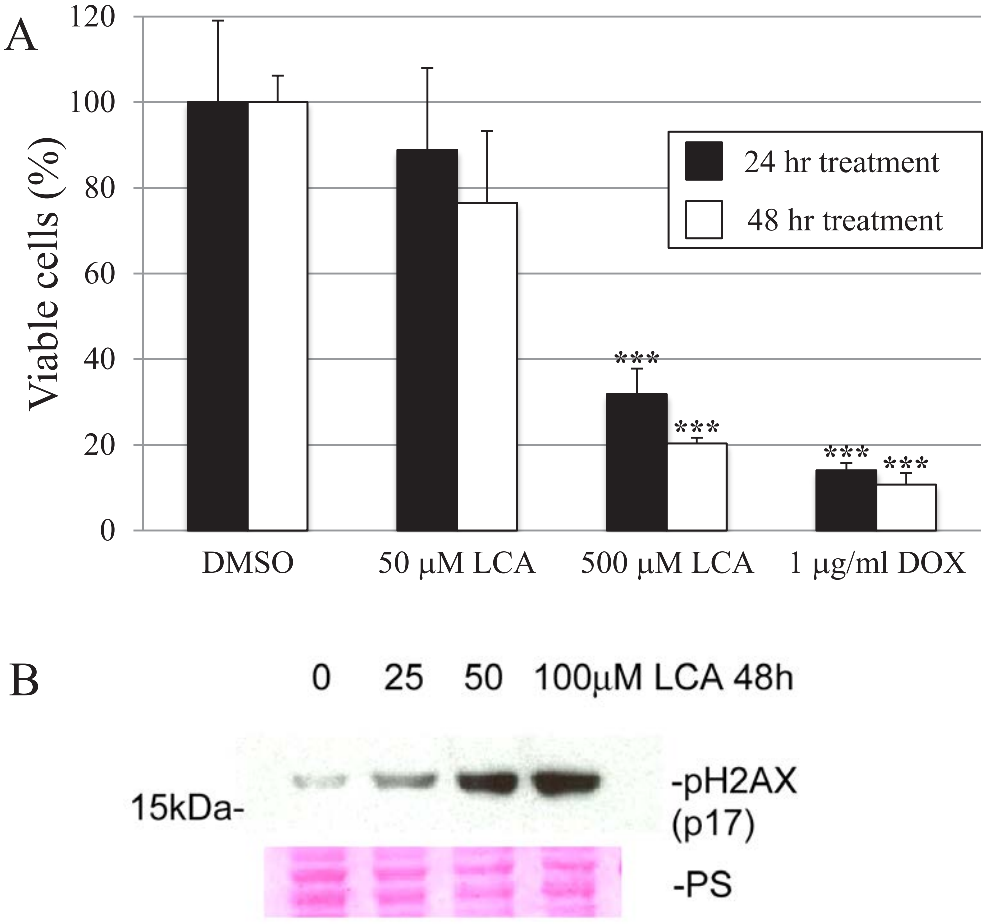

Supplemental Figure 2. LCA kills the drug-sensitive human BC cell line MCF7 and causes DNA damage in MCF cells. A. The percentage of viable MCF cells was calculated as a portion of their population displaying a detectable level of redox potential - which was monitored using the MTT assay as described in Materials and Methods. 1 mg/ml doxorubicin (DOX) was used as a control to demonstrate drug sensitivity of the MCF7 cell line tested. Drug treatments were for either 24 or 48 h. Data are presented as means ± SD (n = 3); ***p<0.001. **B.** In MCF7 cells LCA induces histone H2AX phosphorylation, an early response to DNA damage. Cells were exposed for 24 h to the concentrations of LCA shown. Histone H2AX phosphorylation assay was carried out as described in Materials and Methods. Total protein was stained with Ponceau S (PS) to confirm equal protein load for analysis.

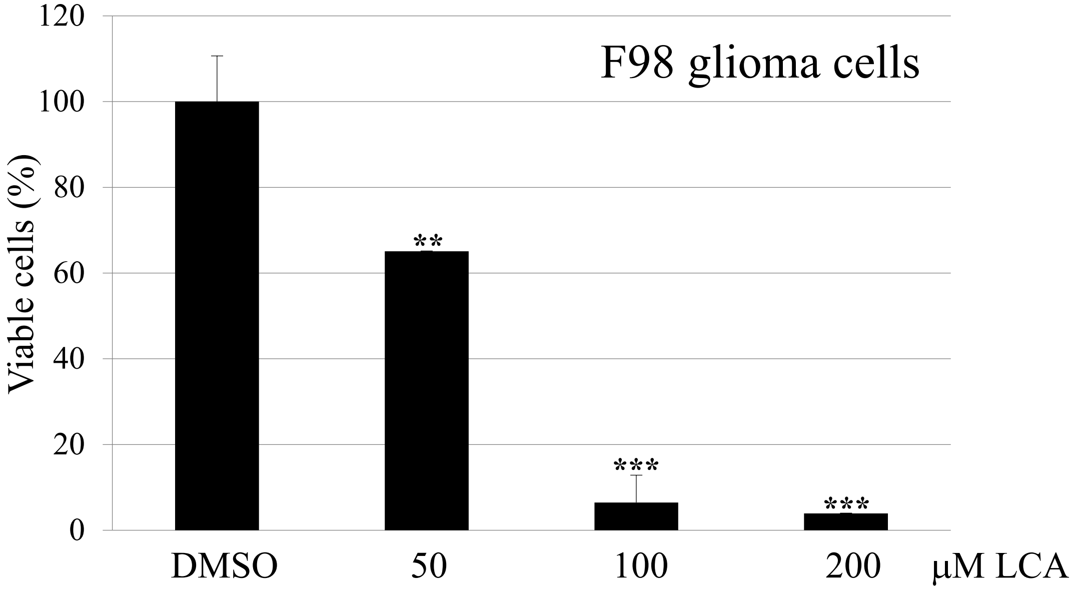

Supplemental Figure 3. LCA kills the cultured rat GL cell line F98. F98 is a drug-resistant line of highly proliferative and invasive cancer cells. Cells were treated with LCA for 24 h. The percentage of viable F98 cells was calculated as a portion of their population displaying a detectable level of redox potential - which was monitored using the MTT assay as described in Materials and Methods. Data are presented as means ± SD (n = 3); **p<0.01; ***p<0.001.
